# A Highly Efficient Composite Catalyst Constructed From NH_2_-MIL-125(Ti) and Reduced Graphene Oxide for CO_2_ Photoreduction

**DOI:** 10.3389/fchem.2019.00789

**Published:** 2019-11-15

**Authors:** Yunxia Zhao, Wei Cai, Jiaxin Chen, Yuanyuan Miao, Yunfei Bu

**Affiliations:** ^1^Jiangsu Collaborative Innovation Center of Atmospheric Environment and Equipment Technology, Nanjing, China; ^2^Jiangsu Key Laboratory of Atmospheric Environment Monitoring and Pollution Control, Nanjing, China; ^3^School of Environmental Science and Engineering, Nanjing University of Information Science and Technology, Nanjing, China

**Keywords:** CO_2_ reduction, photocatalysis, metal-organic frameworks (MOFs), reduced graphene oxide, methyl formate

## Abstract

Substantial consumption of fossil fuels causes an increase in CO_2_ emissions and intensifies global pollution problems, such as the greenhouse effect. Recently, a new type of ultra-low-density porous material, metal-organic frameworks (MOFs), has been developed for the photocatalytic conversion of CO_2_. Herein, a composite photocatalytic catalyst based on NH_2_-MIL-125(Ti) and reduced graphene oxide (rGO@NH_2_-MIL-125) was fabricated through a facile “one-pot” process. The acquired materials were characterized to obtain their structures, morphologies, and optical information. The experimental results showed that methyl formate (MF) was the predominant reaction product, and rGO@NH_2_-MIL-125 exhibited the highest yield of 1,116 μmol·g^−1^·h^−1^, more than twice that of pure MIL-125. The high photoactivity of rGO@NH_2_-MIL-125 can be ascribed to the effective spatial separation and transfer of photoinduced carriers, largely due to the synergistic effect of amino functionality and rGO incorporation. rGO@NH_2_-MIL-125 also displayed acceptable repeatability in cyclic runs for CO_2_ reduction.

## Highlights

- The agglomerate state of NH_2_-MIL-125(Ti) was improved by introducing reduced graphene oxide.
- The composite catalyst could efficiently enhance photogenerated charge separation.
- The composite photocatalyst showed effective activity in reducing CO_2_ in CH_3_OH to HCOOCH_3_.
- The composite catalyst exhibited acceptable stability in cyclic runs for CO_2_ reduction.

## Introduction

With the economic and social development, the CO_2_ concentration in the atmosphere is increasing, causing global warming, and other climate problems. CO_2_ emissions mainly come from mobile or immobile sources (e.g., vehicles and coal power plants). The reduction of CO_2_ emissions is essential to address the global issue of climate change. Both the capture and sequestration technology of CO_2_ (Anwar et al., 2018; Luis Míguez et al., 2018) and the chemical conversion of captured CO_2_ into useful chemicals or clean fuels using solar energy (Fu et al., 2018; Lee et al., 2018; Wang et al., 2018a) at normal temperatures and pressures are promising approaches that can simultaneously solve two problems by addressing global environmental pollution and energy deficiency.

Photocatalytic CO_2_ reduction is one CO_2_ conversion technology, and its key lies in the catalyst. In 1979, Inoue et al. (1979) synthesized semiconductor TiO_2_ as a catalyst for photoirradiation of the CO_2_ reduction reaction in an aqueous solution filled with CO_2_. The reduction products, CH_2_O, HCOOH, CH_3_OH and CH_4_, were obtained, and this result laid the foundation for photocatalytic CO_2_ reduction. Thereafter, TiO_2_ was continuously proven to be an efficient and promising photocatalyst (Liu G., et al., 2018; Wang et al., 2018b, 2019). Various Ti-based modified catalysts have been designed and developed for improving the CO_2_ photoreduction efficiency (Jiang et al., 2018; Zhao et al., 2018). It was found that an appropriate bandgap width and band structure, good electron-hole separation performance, and abundant reaction active sites favor excellent photocatalytic efficiency (Usubharatana et al., 2006; Crake et al., 2017). Metal-organic frameworks (MOFs) are one type of porous material with ordered crystal structures constructed from metal nodes (metal ions) and organic linkers (Millward and Yaghi, 2005). In recent years, research on MOFs has spread to the catalysis and CO_2_ conversion fields (Crake et al., 2017; Fang et al., 2018; Li et al., 2018a; Zhang et al., 2018). Ti-based MOFs are considered promising candidates for CO_2_ photocatalytic reduction (Sun et al., 2014; Li et al., 2018a; Zhang et al., 2018) because their Ti-O clusters can be regarded as isolated titanium oxide quantum dots (Sun and Li, 2017). Fu et al. (2012) and Li et al. (2018a) studied the photocatalytic CO_2_ reduction performance on MIL-125(Ti). After 10 h of UV-light irradiation, 2.41 μmol HCOO^−^ was detected in the acetonitrile (MeCN) solvent with triethanolamine (TEOA). Then, amino-functionalized MIL-125 was also investigated for CO_2_ photoreduction, and a possible mechanism for this photocatalysis reaction was proposed. After introducing the BDC linker with the amine group –NH_2_, the bandgap of MIL-125 was obviously reduced (Fu et al., 2012; Xu et al., 2015), and its optical absorption performance was improved. In addition, the adsorption capability toward CO_2_ was also enhanced by the amino functionality. The HCOO^−^ yield increased to 3.83 μmol.

Even so, the NH_2_-MIL-125 still has a low CO_2_ reduction activity (Xu et al., 2015). To further improve the photocatalytic efficiency, graphene-like two-dimensional materials have often been incorporated into many photocatalysts via covalent or non-covalent interactions (Zhu et al., 2018). A microwave induction platform for the preparation of graphene oxide (GO)-enhanced MOF photocatalysts with highly efficient optical and electronic properties resulted in good photocatalytic oxidation performance and stability for nitric oxide and acetaldehyde (Li et al., 2018b). A strong photocatalytic hydrogen production activity up to ~9.1-fold that of the pure MOF can be achieved by MOF@rGO because the strong π-π interactions between the MOF and rGO can effectively accelerate the electron–hole pair separation (Karthik et al., 2018).

Herein, a NH_2_-MIL-125(Ti) and reduced graphene oxide (rGO) composite was synthesized *in situ* by a facile “one-pot” method as an efficient photocatalyst for CO_2_ reduction. The bare MIL-125(Ti) was also prepared to compare with NH_2_-MIL-125(Ti) in order to highlight the advantage of –NH_2_ based on its superior optical performance. rGO with a two-dimensional structure was expected to be a superior charge transfer medium due to its fast light transmission, outstanding conductivity and high carrier mobility (Bao and Chen, 2018). Moreover, rGO was also supposed to play a part in the structural adjustment of NH_2_-MIL-125(Ti). Therefore, the objective catalyst was compared to pure MIL-125(Ti) and NH_2_-MIL-125(Ti) to ascertain the synergistic effect of the –NH_2_-containing linker and incorporated rGO. The photocatalytic reaction here was conducted in a catalyst-methanol slurry system at normal atmospheric pressure and temperature. MeCN, TEOA and other toxic organic solvents were avoided for use as hole scavengers. Instead, hypotoxic methanol was chosen as the reducing agent due to its high CO_2_ absorption capacity.

## Experimental

### Synthesis

Pure MIL-125(Ti) was prepared through a solvothermal process referenced in the literature (Yang et al., 2017; Rahmani et al., 2018; Wang et al., 2018c). Typically, titanium isopropoxide (1.5 mmol) and H_2_BDC (6 mmol) were dissolved in a mixed solvent of DMF and CH_3_OH (18 mL/2 mL) under magnetic stirring for 0.5 h. Then, the solution was transferred to a Teflon-lined stainless-steel autoclave and heated at 150°C for 72 h. After being filtered, the white solid product was washed thrice with DMF and CH_3_OH, respectively. During the washing process, the suspension was subjected to natural precipitation and removal of the supernatant. Finally, the MIL-125(Ti) powder was activated under vacuum drying at 80°C for 10 h.

Amino-substituted titanium MOF NH_2_-MIL-125(Ti) was also prepared by a similar procedure except that H_2_BDC was replaced by H_2_BDC-NH_2_. Finally, a yellow solid powder was obtained.

The NH_2_-MIL-125(Ti) and rGO composite was synthesized according to a similar solvothermal process. First, a certain amount of GO power was dispersed into 10 mL DMF by ultrasonication for 1 h. The amount of GO added was 5 wt. % of the parent MOF. In the meantime, titanium isopropoxide (1.5 mmol) and H_2_BDC (6 mmol) were dissolved in a mixed solvent of DMF and CH_3_OH (18 mL/2 mL). Then, the above two were mixed together, stirred constantly for 1 h, and subjected to a solvothermal process at 150°C for 72 h. Usually, this solvothermal condition reduces GO to rGO (Yang et al., 2016a). The following procedures were the same as those for the above two pristine MOFs. The obtained gray powder was labeled as rGO@NH_2_-MIL-125.

### Characterization

The structures of all three obtained samples were measured by X-ray diffraction (XRD) (XD-3, Purkinjie), scanning electron microscopy (SEM) (S4800, Hitachi) and N_2_ absorption-desorption analysis (ASAP 2020, Micromeritics). Fourier transform infrared spectra (FT-IR) were obtained on a MB-154S infrared spectrometer (Bomen, Canada) to investigate the samples' chemical groups. The optical properties of all samples were examined by ultraviolet-visible diffuse reffectance spectroscopy (DRS) (UV-2600, Shimadzu) and photoluminescence spectroscopy (PL) (F-4500, Hitachi) with a 325 nm excitation wavelength. The photoelectrochemical measurements were performed in a CHI760 electrochemical workstation using a three-electrode electrochemical cell. A Pt wire and a Ag/AgCl electrode (3 M KCl) served as the counter and reference electrodes, respectively. The electrolyte was 0.5 M Na_2_SO_4_. The working electrode was FTO glass, and the sample was deposited on it by a drop casting method (Wang et al., 2018d). Five milligrams of photocatalyst powder was dispersed in 1 mL of ethanol containing 10 μL of naphthol by ultrasonication (1 h). Then, two drops of the sample solution were scattered on the FTO glass, and the coated area was fixed at 1 cm^2^. After being dried at 60°C for 12 h in a vacuum oven, a homogeneous film was obtained.

### Photocatalytic Activity Test

The photocatalytic activity tests of all the synthesized samples were carried out using a photochemical reaction instrument (BL-GHX-V, BiLon, China). First, pure CO_2_ gas was bubbled into the catalyst-CH_3_OH system (30 mg/30 mL) for saturation in a 50 mL quartz tube, which was used as the photocatalytic reactor. Then, the quartz tube was sealed, and a magnetic stirrer provided ideal mixing at the bottom. A 250 W high-pressure mercury lamp served as a light source and illuminated the photocatalytic reactor from the side. Finally, the reactant-product mixed solution was analyzed by gas chromatography (GC 9790II, Fuli, China), and the photocatalytic efficiency was assessed by the yield of the product methyl formate (MF).

## Results and Discussion

### Catalyst Characterization

#### Structural Properties

**Figure 1:**
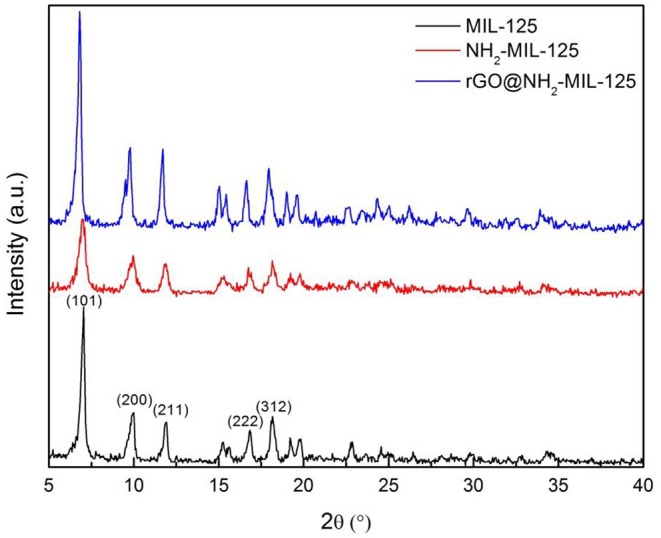
The XRD patterns of the three acquired catalysts.

The XRD patterns for the pure MOFs MIL-125, NH_2_-MIL-125, and the composite rGO@NH_2_-MIL-125 are presented in Figure 1. The strong diffraction peaks of MIL-125 at 6.8°, 9.5°, 11.6°, 16.6°, and 18.0° can be indexed to the (101), (200), (211), (222), and (312) planes, respectively (Kim et al., 2013; Wang et al., 2016; Yuan et al., 2016; Yang et al., 2017; Rahmani et al., 2018; Han et al., 2019) of its orthorhombic crystal structure. The XRD pattern of as-prepared NH_2_-MIL-125 is in good agreement with other reports (Kim et al., 2013; Guo et al., 2015; Li et al., 2018b). The pattern also exhibits characteristic peaks similar to those of MIL-125, suggesting a similar crystal structure. The rGO@NH_2_-MIL-125 composite possesses diffraction peaks similar to those of NH_2_-MIL-125 but with higher intensity. This result demonstrates that NH_2_-MIL-125 crystals were perfectly formed in the presence of rGO. Notably, no obvious typical rGO peaks (Yuan et al., 2016) are shown in the XRD pattern of the rGO@NH_2_-MIL-125 sample due to the low amount and relatively low diffraction intensity of the rGO component (Yang and Xu, 2013) in comparison with the MOFs. It is interesting to discover that the diffraction peaks of rGO@NH_2_-MIL-125 shift to the left relative to those of NH_2_-MIL-125, which indicates strong interactions between the rGO and MOF components in the rGO@NH_2_-MIL-125 composite. This phenomenon was also observed by Li et al. (2018b).

**Figure 2:**
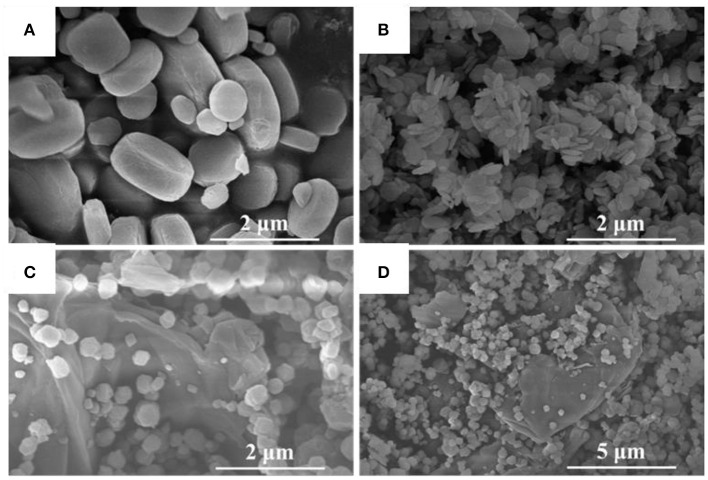
SEM images of MIL-125 **(A)**, NH_2_-MIL-125 **(B)**, and rGO@NH_2_-MIL-125 **(C,D)**.

The morphological details of MIL-125, NH_2_-MIL-125 and the composite rGO@NH_2_-MIL-125 measured by SEM are shown in Figures 2A–D. MIL-125 has a tablet-like form and smooth surface with an average particle size of 1 μm. NH_2_-MIL-125 exhibits a similar shape as MIL-125, but it has a much smaller size in diameter or thickness, which coincides with the results of XRD patterns. The smaller the crystal size, the wider the diffraction peak. In Figure 2B, it can be seen that these much smaller tablets aggregate together in piles. Obviously, the dispersion of MIL-125 particles is better than that of NH_2_-MIL-125. From the SEM images of rGO@NH_2_-MIL-125, it can be clearly seen that the two components are intertwined with each other. rGO has a wrinkled layered structure, and NH_2_-MIL-125 maintains a polyhedron structure loaded on the surface of rGO. However, the shape of NH_2_-MIL-125 particles in the composites becomes irregular due to the incorporation of rGO, as seen in the higher magnification SEM images (Figure S1). Compared with Figure 2B, the dispersion of MOF particles in Figures 2C,D is better, which demonstrates that aggregation could be avoided by rGO.

**Figure 3:**
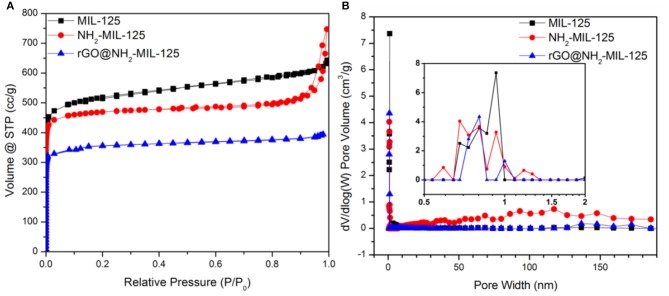
Nitrogen adsorption–desorption isotherms **(A)** and the corresponding pore size distribution curves **(B)** of MIL-125, NH_2_-MIL-125 and rGO@NH_2_-MIL-125.

N_2_ physisorption experiments were conducted to obtain the porous structures and specific surface areas of the samples. The as-prepared samples are all type I isotherms, as shown in Figure 3A, indicating their microporous structures, which were verified by the pore size distribution curves in Figure 3B. The corresponding calculated parameters are listed in Table 1. As shown, MIL-125, NH_2_-MIL-125 and rGO@NH_2_-MIL-125 display Brunauer-Emmett-Telle (BET) surface areas of 1,669, 1,490, and 1,048 m^2^·g^−1^, respectively. A higher surface area can provide more active sites for photocatalytic reactions. rGO@NH_2_-MIL-125 possesses a smaller surface area and micropore volume than NH_2_-MIL-125. Here, the intended content of GO added in the composite was 5 wt.%. However, the surface area of the rGO@NH_2_-MIL-125 composite obtained was much less than the value on a prorata calculation. Therefore, the introduction of rGO affected the structure of NH_2_-MIL-125 particle agglomerates and decreased their surface area and pore volume. As seen from the pore size distribution curves, more mesopores exist in NH_2_-MIL-125 based on the hysteresis loop observed in its N_2_ adsorption-desorption isotherm. This difference may be caused by its aggregated clusters, and thus, NH_2_-MIL-125 exhibits a larger total pore volume than MIL-125 and a similar micropore ratio. When rGO is incorporated into the reaction precursor, the obtained rGO@NH_2_-MIL-125 exhibits a lower mesopore volume and a more centralized pore width distribution, which is in agreement with its higher dispersibility relative to those of NH_2_-MIL-125.

**Table 1:** Specific surface areas and pore structure parameters of the three as-prepared samples.

**Samples**	***S*_**BET**_/m^**2**^**·g**^**−1**^**	***V*_**t**_/cm^**3**^**·g**^**−1**^**	***V*_**micro**_/cm^**3**^**·g**^**−1**^**	***V*_**micro**_/*V*_**t**_**
MIL-125	1,669	0.99	0.62	63%
NH_2_-MIL-125	1,490	1.15	0.64	56%
rGO@NH_2_-MIL-125	1,048	0.71	0.55	77%

### Chemical Composition

**Figure 4:**
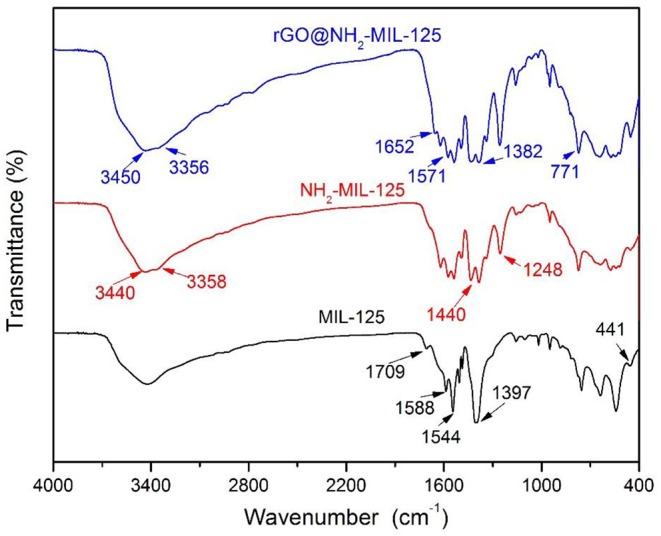
FTIR spectra of the three acquired catalysts.

Figure 4 shows the comparison of the FTIR spectra of the pure MOFs MIL-125 and NH_2_-MIL-125 and the composite rGO@NH_2_-MIL-125. In the FTIR spectrum of MIL-125, the large broad adsorption band in the range of 3,200~3,700 cm^−1^ is attributed to free solvent molecules in the framework pores (Yang et al., 2017). The strong bands at 1,397~1,709 cm^−1^ are assigned to the vibrations of carboxylate groups, which belong to the linkers in the framework of MIL-125 (Martis et al., 2013; Wang et al., 2015; Yang et al., 2017, 2018). The characteristic absorption of benzene rings is observed in the range of 800~1,200 cm^−1^, and the O-Ti-O vibrations are located in the range of 400~800 cm^−1^ (Yang et al., 2017, 2018). NH_2_-MIL-125 possesses three similar main absorption regions as MIL-125 except the characteristic stretching vibrations of the hydroxyl at 3,440 cm^−1^ and the amino at 3,358 cm^−1^ (Martis et al., 2013; Wang et al., 2015; Li et al., 2018b). In addition, the strong band at 1,248 cm^−1^ is assigned to the stretching vibrations of C-N from the aromatic amine (HadŽi and Škrbljak, 1957). rGO@NH_2_-MIL-125 shows almost the same adsorption bands as NH_2_-MIL-125. The absence of GO-related stretching bands for oxygen-containing functional groups confirms the effective reduction of GO into rGO under the solvothermal reaction process. In addition, a new absorption band originating from the graphene skeletal vibration appears at 1,652 cm^−1^, which is shifted from its common band position of 1,630 cm^−1^ (Al Nafiey et al., 2017; Zhao et al., 2017). This shift also hints at the interactions between rGO and MOF components in the rGO@NH_2_-MIL-125 composite.

### Optical Properties

**Figure 5:**
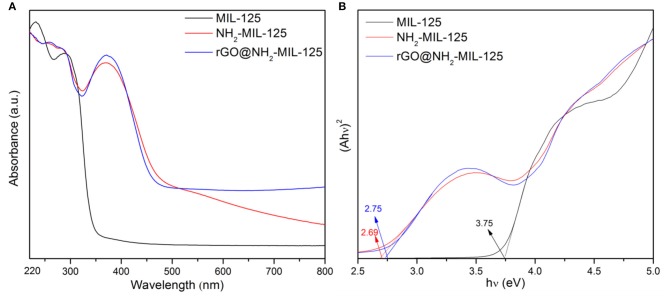
**(A)** UV-vis diffuse reflectance absorption spectra of the three obtained samples and **(B)** plots of (Ahν)^2^ versus the photon energy.

The optical absorption characteristics of pure MIL-125, NH_2_-MIL-125 and the composite rGO@NH_2_-MIL-125 were investigated by UV-vis DRS in Figure 5A. For MIL-125, its absorption peaks are in the UV-light range, while the pure NH_2_-MIL-125 and the composite rGO@NH_2_-MIL-125 absorb light from the UV to visible-light range, which can improve the utilization of solar energy and produce more electron–hole pairs. For MIL-125, the O to Ti ligand-to-metal charge transfer in TiO_5_(OH) inorganic clusters was confirmed by Sun and Li (2017) and Kim et al. (2013), and H_2_BDC-NH_2_ or rGO apparently influences the change transfer phenomena in these TiO_5_(OH) clusters in NH_2_-MIL-125 or rGO@NH_2_-MIL-125. Moreover, rGO@NH_2_-MIL-125 possesses a higher absorption intensity than NH_2_-MIL-125 at wavelengths >500 and 339~411 nm, indicating that the addition of rGO may positively affect the optical properties. In Figure 5B, the bandgap values can be determined from the plots of (Ahν)^2^ vs. the photon energy (hν) by extrapolating the maximum slope to the x axis (Li et al., 2018b; Wu et al., 2018). For semiconducting MOFs, the bandgap is defined as the gap between the highest occupied molecular orbital (HOMO) and the lowest unoccupied molecular orbital (LUMO) according to the molecular orbital method (Maina et al., 2017; Li et al., 2018a). Thus, the HOMO-LUMO gap of MIL-125 is 3.75 eV and that for NH_2_-MIL-125 is 2.69 eV, which is very close to that of the composite rGO@NH_2_-MIL-125 (2.75 eV). Thus, the introduction of the amino into the BDC linker can clearly reduce the HOMO-LUMO gap value of MIL-125. The photocatalyst with a higher HOMO-LUMO gap requires light with higher energy to generate photoinduced electrons and holes. Obviously, amino groups introduced into the linkers of MIL-125 are favorable for extending the optical adsorption properties and decreasing the photodriving force.

**Figure 6:**
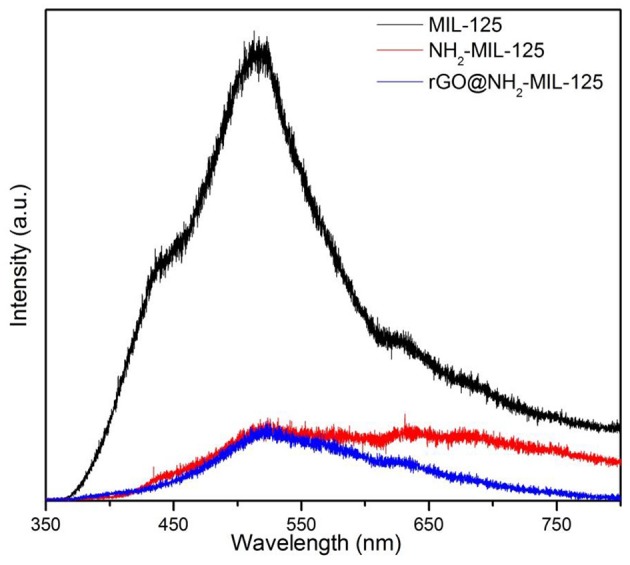
PL spectra measured at room temperature for the three as-prepared photocatalysts.

The trapping and lifetime of photogenerated charges has a great effect on the photocatalytic activity of a semiconductor photocatalyst (Liu G., et al., 2018; Liu H., et al., 2018). The PL spectra of the three catalysts are shown in Figure 6 since PL is usually used for evaluating the recombination of photoinduced charge carriers. The pristine MIL-125 displays a strong peak at ~525 nm. However, the PL intensities of pure NH_2_-MIL-125 and the composite rGO@NH_2_-MIL-125 were significantly weakened. At the optimum wavelength of 525 nm, they are very close. However, in the higher wavelength scope, the intensity of rGO@NH_2_-MIL-125 is even lower. Generally, the low PL intensity can result from a low recombination rate of photogenerated electron-hole pairs (Huang et al., 2018), suggesting the longer service life of the electron hole. Thus, both the amino-functionalized linker and the rGO incorporation are favorable for the mobility of photoexcited electrons and prohibiting photoinduced charge carrier recombination.

**Figure 7:**
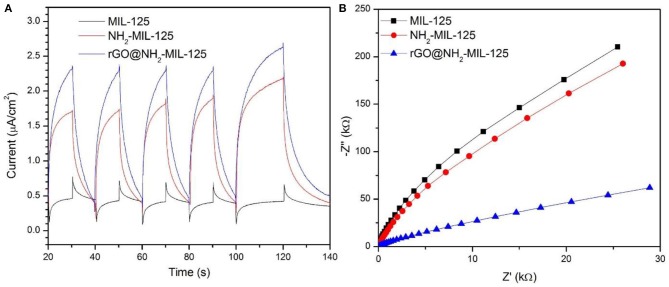
**(A)** Photocurrent potential curves and **(B)** EIS Nyquist plots of the three as-prepared catalysts.

Photoelectrochemical measurements were performed to further study the charge separation and transfer efficiency of all catalysts. In Figure 7A, the three as-prepared samples display basically reproducible photocurrent responses during intermittent visible-light irradiation with the same time intervals. Clearly, the photocurrent densities decreased following the sequence of rGO@NH_2_-MIL-125, NH_2_-MIL-125, and MIL-125. A high photocurrent density is attributed to a high separation efficiency of photogenerated electron-hole pairs (Wan et al., 2017a,b). Thus, both amino groups containing linkers and rGO benefit the efficient separation of photoinduced e^−^ and h^+^ for further improvement of enhancing the photocatalytic activity of MIL-125. The electrochemical impedance spectroscopy (EIS) test is another effective way to analyse the charge transfer properties. In theory, a smaller arc radius in the EIS Nyquist plot indicates a lower charge transfer resistance and represents a higher migration and transfer efficiency of photoexcited electron-hole pairs (Wan et al., 2017b; Wang et al., 2018d). In Figure 7B, the introduction of rGO and amino groups in MIL-125 causes a significant decrease in the arc radius, implying the lowest charge transfer resistance on the surface of the electrode with rGO@NH_2_-MIL-125, which is consistent with the photocurrent results.

### Photocatalytic CO_2_ Reduction Performance

**Figure 8:**
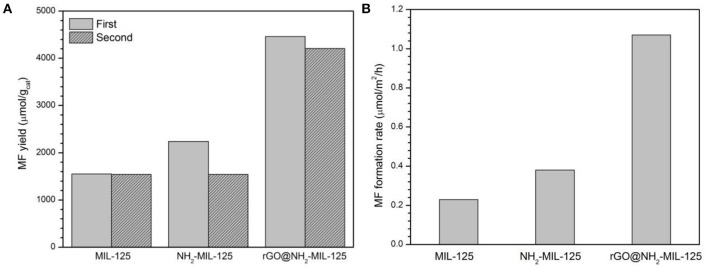
MF yield in units of μmol·${\text{g}}_{\text{cat}}^{-1}$ **(A)** and in units of μmol·m^−2^·h^−1^ **(B)** with various catalysts for CO_2_ photoreduction in CH_3_OH.

Figure 8A shows the photocatalytic activity of MIL-125, NH_2_-MIL-125 and rGO@NH_2_-MIL-125. These three materials were used to reduce CO_2_ in CH_3_OH after 4 h of light irradiation, and the predominant reaction product was MF. Since the light source was UV-light, the photon energy was enough to excite all the three photocatalysts. For the fresh samples, the data in the figure show that NH_2_-MIL-125 has a slightly higher MF yield than MIL-125, although the former possesses a lower BET surface area. This difference might be related to the special structure and functional groups of NH_2_-MIL-125. Excluding the surface area, the MF formation rate in units of μmol·m^−2^·h^−1^ is shown in Figure 8B. This result for NH_2_-MIL-125 is superior to that of MIL-125, which is attributed to its more effective separation of photoinduced charge carriers. Among these three samples, rGO@NH_2_-MIL-125 exhibits the highest yield of MF, which is 1,116 μmol·g^−1^·h^−1^ and more than twice that of pure MIL-125. This is also more than that of metal sulfides (Chen et al., 2013a,b, 2015). In addition, the honeycomb lattice of rGO allows electrons to pass rapidly. It has good electron conductivity and results in the superior catalytic performance of rGO@NH_2_-MIL-125. Thus, the high photoactivity of rGO@NH_2_-MIL-125 can be ascribed to the effective spatial separation and transfer of photoinduced carriers, largely due to the synergistic effect of amino functionality and rGO incorporation.

The catalysts were recovered from the photocatalytic suspension system by centrifugation after each use. In Figure 8A, the repeatability of the NH_2_-MIL-125 series sample is worse than that of MIL-125, which indicates the better stability of MIL-125. According to the XRD patterns of fresh and used samples after two cycles (Figure S2), there is no drastic change on their crystal structure. Only the intensities of XRD patterns of used catalysts are decreased, which can be explained by some adsorbed species in them (Sadeghi et al., 2018). The FTIR spectra of the used samples (Figure S3) show some new bands at 2,800~2,900 cm^−1^ and 3,500~3,600 cm^−1^ from CH_3_OH (Forester and Howe, 1987), indicating that the adsorbed specie was methanol in the recycled catalysts. To further investigate the recyclability of rGO@NH_2_-MIL-125 with the best photocatalysis activity, four times cycling tests of photocatalytic CO_2_ reduction over it were conducted. As shown in Figure S4, the yields of MF are still above 85% after four cycles, which indicates acceptable recyclability of GO@NH_2_-MIL-125 for photocatalytic CO_2_ reduction. In the future, the stability of the photocatalysts in cyclic runs is expected to improve by optimizing the recycling operations.

**Figure 9:**
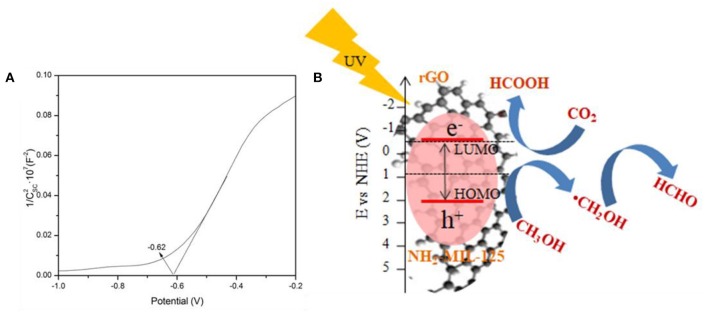
**(A)** Mott-Schottky (MS) plots of NH_2_-MIL-125 and **(B)** schematic of the possible mechanism of photocatalytic CO_2_ reduction over rGO@NH_2_-MIL-125 catalysts.

To clarify the possible mechanism for the photocatalytic CO_2_ reduction in CH_3_OH solution over rGO@NH_2_-MIL-125 catalysts under UV-light irradiation, the band structure of NH_2_-MIL-125 was investigated by Mott–Schottky measurements, and the Mott-Schottky plot is shown in Figure 9A. The positive slope of the plot indicates that NH_2_-MIL-125 is an n-type semiconductor (Yang et al., 2016a). If NH_2_-MIL-125 is set as a conventional semiconductor, the X-axis intercept of −0.62 of the linear region can be considered its flat band potential (V_fb_) with the unit V vs. Ag/AgCl. According to the equation (Ao et al., 2018; Cai et al., 2018; Wang et al., 2018d), *E* (NHE, normal hydrogen electrode) = *E*(Ag/AgCl) + 0.1976 V, the converted potential NHE is obtained. Since V_fb_ is generally 0.2 V more positive than the conduction band (CB) potential (E_CB_) for an n-type semiconductor (Chen et al., 2013a; Wang et al., 2018d), the calculated E_CB_ is ~-0.62 V vs. NHE. Based on the corresponding LUMO potential of NH_2_-MIL-125 (−0.62 V) (Zhao et al., 2019) and the bandgap (HOMO–LUMO gap width) in Figure 5A, the position of the HOMO energy level is further estimated to be 2.07 V vs. NHE. Under UV-light irradiation, NH_2_-MIL-125 can absorb photon energy and then be excited to generate electron-hole pairs. The electrons in the LUMO of NH_2_-MIL-125 transfer to the rGO surface due to its excellent electron mobility (Li et al., 2018c; Zhu et al., 2018), while the holes can remain on the HOMO of NH_2_-MIL-125 since the recombination of photogenerated charge carriers is greatly inhibited. As reported by Wan et al., (2017a), rGO itself doesn't contribute to the generation of electrons and holes under light irradiation. It only serves as an electron conductor medium. Thus, the introduction of rGO offers a greater range of carrier motion, which can efficiently suppress the recombination of photoinduced electrons and holes as well as increase the ability of electronic transmission. A possible mechanism is proposed, as displayed in Figure 9B, according to the above HOMO–LUMO structure. The HOMO of NH_2_-MIL-125 is more positive than the reported *E*_0_*(CH*_3_*OH/*·*CH*_2_*OH)* (0.927 V) (Qin et al., 2011; Yang et al., 2016b; Chen et al., 2017; Ao et al., 2018), so the photoinduced holes can oxidize CH_3_OH into HCHO with H^+^ formation. The LUMO of NH_2_-MIL-125 is negative than *E*_0_*(CO*_2_*/HCOOH)* (−0.61 V) (Qin et al., 2011; Yang et al., 2016b; Chen et al., 2017; Ao et al., 2018) and can reduce CO_2_ into HCOOH in the presence of H^+^. Finally, MF can be produced not only via the esterification of HCOOH and CH_3_OH but also by the dimerization of HCHO based on the Tishchenko reaction (Qin et al., 2011; Yang et al., 2016b; Chen et al., 2017).

## Conclusions

The composite rGO@NH_2_-MIL-125 was fabricated *in situ* through a “one-pot” solvothermal process. The agglomerated state of NH_2_-MIL-125(Ti) was improved by introducing rGO. Its photocatalytic activity was evaluated by the photocatalytic CO_2_ reduction in CH_3_OH solution and compared with those of the other two prepared samples MIL-125 and NH_2_-MIL-125. The results showed that MF was the predominant reaction product, and rGO@NH_2_-MIL-125 exhibited the highest yield of 1,116 μmol·g^−1^·h^−1^, which is two times more than that of pure MIL-125. The effective separation of photogenerated electrons and holes largely due to the synergistic effect of amino functionality and rGO incorporation contributed to the high photoactivity of rGO@NH_2_-MIL-125. It also displayed acceptable repeatability in cyclic runs for CO_2_ photocatalytic reduction. However, its repeatability without achieving the largest value indicates that optimization of the recovery processes in future research can further improve the stability of the photocatalysts in cyclic runs.

## Data Availability Statement

The datasets generated for this study are available on request to the corresponding author.

## Author Contributions

YZ did the experiment, analyzed the data and drafted the article. WC collected data and analyzed them. JC and YM assisted the work of YZ and WC. YB designed the work.

### Conflict of Interest

The authors declare that the research was conducted in the absence of any commercial or financial relationships that could be construed as a potential conflict of interest.
