# Supplementary material for: A Highly Efficient Composite Catalyst Constructed From NH2-MIL-125(Ti) and Reduced Graphene Oxide for CO2 Photoreduction
Source: Front Chem. 2019 Nov 15;7:789. doi: 10.3389/fchem.2019.00789 (PMC6873613; doi:10.3389/fchem.2019.00789)
Supplement: Supplementary file 1 [file Presentation_1.pdf]

Supplementary Information

A Highly Efficient Composite Catalyst Constructed from NH_2-MIL-125(Ti) and Reduced Graphene Oxide for CO_2 Photoreduction

Yunxia Zhao [1,2,3], Wei Cai [1,2,3], Jiaxin Chen[3], Yuanyuan Miao[3], Yunfei Bu[1,2,3]*

[1] Jiangsu Collaborative Innovation Center of Atmospheric Environment & Equipment Technology, Nanjing, China, [2] Jiangsu Key Laboratory of Atmospheric Environment Monitoring and Pollution Control, Nanjing, China, [3] School of Environmental Science and Engineering, Nanjing University of Information Science & Technology, Nanjing, China

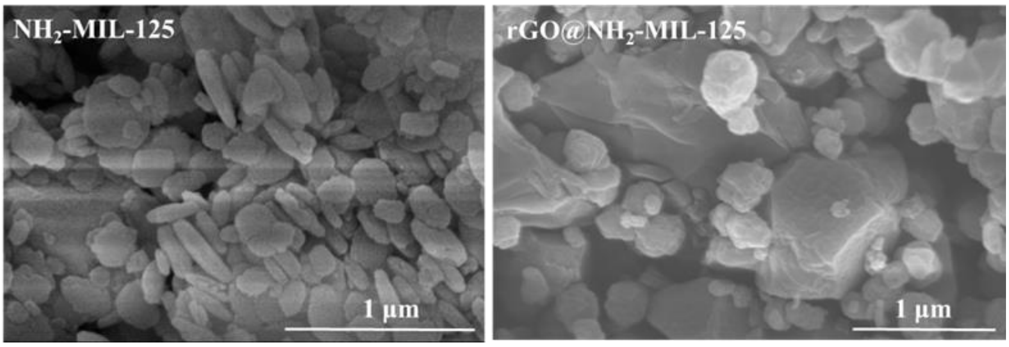

Fig. S1. Higher magnification SEM images.

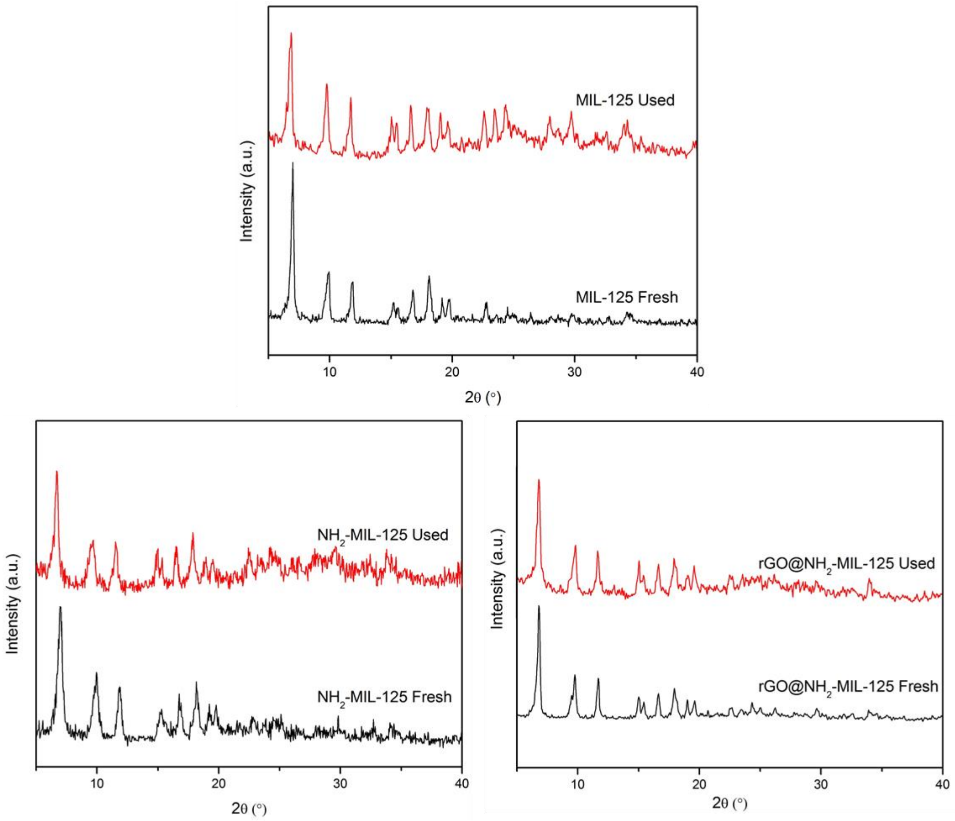

Fig. S2. XRD patterns of the fresh and used samples after two cycles.

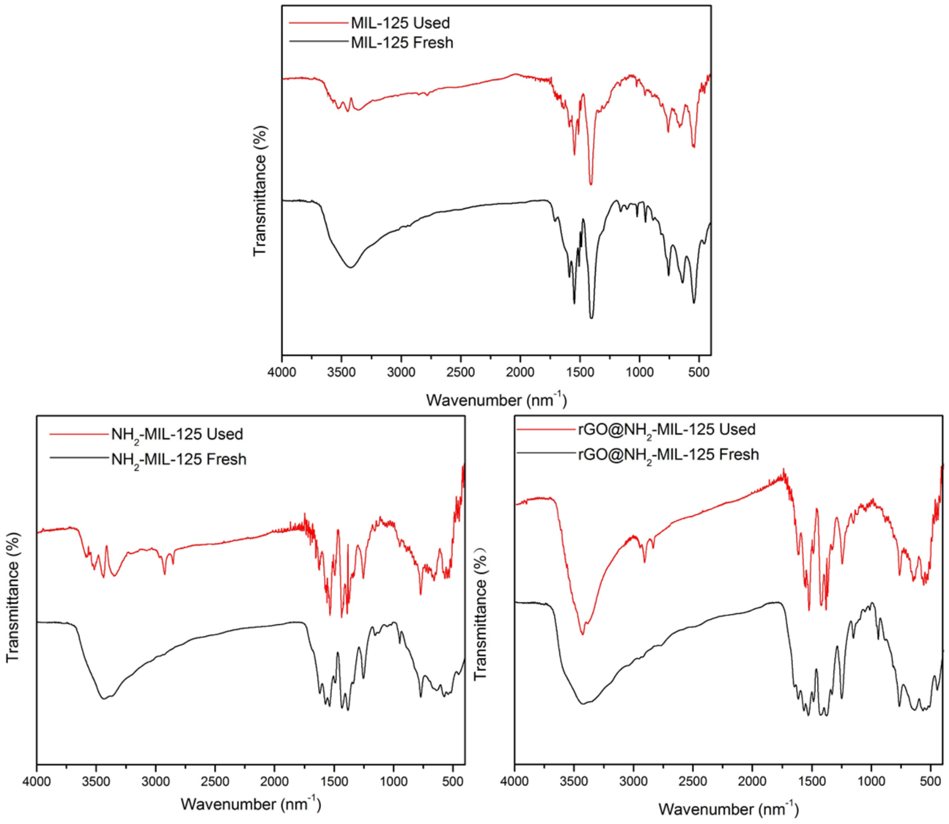

Fig. S3. FTIR spectra of the fresh and used samples after two cycles.

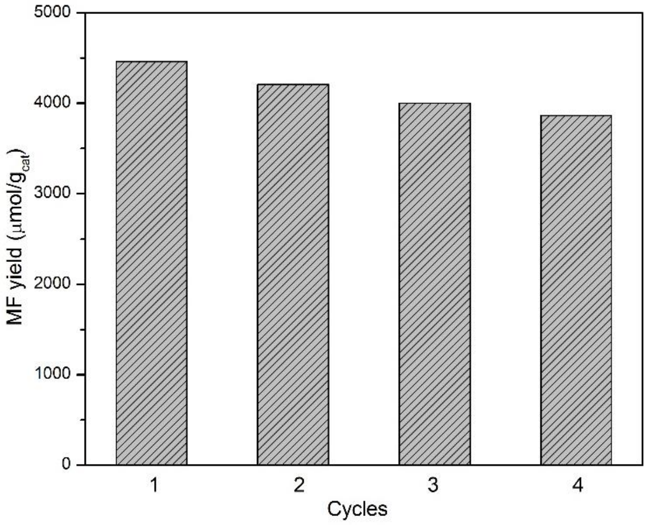

Fig. S4. Recycling tests of photocatalytic CO_2 reduction over rGO@NH_2-MIL-125.
